# Primary School Children’s Sleep Habits: Association with Socioeconomic Factors and Physical Activity Habits

**DOI:** 10.3390/children9070965

**Published:** 2022-06-28

**Authors:** Anabela Afonso, Gonçalo Jacinto, Paulo Infante, Teresa Engana

**Affiliations:** 1 CIMA, IIFA, University of Évora, 7000-671 Évora, Portugal; gjcj@uevora.pt (G.J.); pinfante@uevora.pt (P.I.); 2 Department of Mathematics, ECT, University of Évora, 7000-671 Évora, Portugal; 3 Youth and Sport Division, Évora City Council, 7004-506 Évora, Portugal; teresaengana@cm-evora.pt

**Keywords:** child health, children’s sleep habits questionnaire, logistic regression, parent report, sleep habits

## Abstract

Sleep disorders have significant health impacts and affect children’s performance and wellbeing. This study aims to characterise the sleep habits of Portuguese primary school children considering socioeconomic factors, daily lifestyle, presence of electronic devices in the bedrooms, and the practice of physical and sports activity (PSA) and to identify clinical factors that may be related to a child’s insufficient sleeping time. This study involved 1438 primary students. Sleep habits and problems were assessed using the short version of the Children’s Sleep Habits Questionnaire (CSHQ). The mean sleep duration was 9 h 37 min/ per night (SD = 44 min). The prevalence of global sleep disturbances was 25.8%. The main factors we identified to be positively associated with a child’s sleep deficit (i.e., <10 h) were: being older than 7 years, living further away from school, having electronic devices in the bedroom, going to bed and eating dinner later, less daily time of PSA, and having higher scores on the sleep duration subscales. Sleeping and eating habits, electronic devices in the bedroom, and a low level of engagement with PSA are associated with children’s sleep disturbance and shorter sleep duration.

## 1. Introduction

Adequate sleep is a determining factor for health, especially for children (under ten years old) [1]. Sleep problems take on a global dimension. According to the American Academy of Sleep Medicine (AASM), school-age children (age 6–12 years) should sleep 9 to 12 h per day on a regular basis to promote optimal health [2]. However, some studies in countries including Portugal, Brazil, the Netherlands, Spain, the United States of America, China, and Japan show that children sleep less than the recommended amount [3]. Sometimes, the responsible adult for the child is not aware that the child has sleep problems, since their reports provide information different from objective sleep assessment measures [4].

Sleep disorders have negative effects on cognitive functions, school performance, and emotional and behavioural regulation and are associated with obesity, among other problems [5,6,7,8,9,10,11,12]. On the other hand, the reduced practice of physical activity and sports, the screen behaviour and electronic media use (mobile devices, television, video games, and the internet) are factors that have been associated with sleep disorders in children [13,14,15]. However, there are some inconsistent results among studies about the effects of age, sex, and socioeconomic characteristics on children’s sleep-related habits [16]. These differences are due to physical, environmental, economic, social, and cultural factors [17,18].

There are few published studies about the sleep habits of Portuguese children obtained with validated questionnaires. Most of the studies were conducted in Portuguese urban pediatric populations [19,20,21,22]. In [23], a sample of children from medium-high and low population density areas was analysed. No significant differences were found in the overall prevalence of sleep problems (reported by parents), nighttime sleep duration, and total daily sleep between medium-high and low population density areas.

To our knowledge, there are no studies that have analyzed the relationship between sleep habits, sleep quantity, socioeconomic factors, and physical activity. Therefore, this study aims to explore these relationships in primary school children (i.e., first to fourth grade) living in a low population density area of Portugal. We assess whether bedtime and wake time, sleep duration, and sleep problems are associated with children’s sex, school location, dinner time, bedroom environment, and their involvement in physical activity and sports (PSA). We identify some factors associated with insufficient sleep time. Following the AASM recommendation and based on the empirical experience of the pediatrician in the research group, it was defined that children with less than 10 h of sleep would have insufficient sleep.

## 2. Materials and Methods

### 2.1. Participants

The data in this paper are from a study conducted on the sleep habits and physical and sports activity of children in the first four years of primary school (typically ages 6 to 10 years) in the municipality of Évora in 2019.

The invitation to participate in the study was through flyers and consent forms. These were distributed to the children in classrooms to be given to the person responsible for their education. The questionnaires were distributed to all four groups of schools in the municipality of Évora, i.e., 22 schools in total.

The Ethics Committee of the University of Évora gave a favourable opinion of the study. In addition, it was approved by the Directorate-General for Education of Portugal.

A total of 2194 questionnaires were distributed and 1474 were returned (a response rate of 67%). Three questionnaires were excluded because they presented filling errors, and 33 were discarded because it was impossible to calculate at least half of the CSHQ-PT subscales. Thus, the final sample was composed of 1438 questionnaires.

### 2.2. Instrument

We used two questionnaires, both completed by parents/the person responsible for the child’s education, to collect information about children in primary school:

1. The first questionnaire had questions about the characteristics of the children, the person in charge of their education and their degree of kinship, the distance from the home to the school, an individual or shared bedroom, whether electronic devices were present in the bedroom, whether the children accumulated at least 60 min of PSA per day (in the playground, school-based physical activity, physical activities, …), whether the children belongs to a sport association, the monthly expenditure on PSA, and a question to the person in charge of the child’s education about their opinion of whether PSA helps children to sleep better.
2. The second was the validated Portuguese short version of the Children’s Sleep Habits Questionnaire (CSHQ-PT) [24,25]. This instrument was a 33-item parent-report that assesses the sleep habits and disorders of the school-aged children within the last week or a “typical” recent week. Items were rated on a three-point scale; usually (5 to 7 times/week), sometimes (2 to 4 times/week), and rarely (0 to 1 time/week), and the score of some items was reversed. For the two last items, the three-point response options were not sleepy, very sleepy, and falls asleep. The items were grouped into eight subscales: bedtime resistance (6 items), sleep onset delay (1 item), sleep duration (3 items), sleep anxiety (4 items), night wakings (3 items), parasomnias (7 items), sleep-disordered breathing (3 items), and daytime sleepiness (8 items). The sleep disturbance index (SDI) corresponded to the sum of the subscale scores, and a higher score corresponded to more disturbed sleep. Based on previous studies of Portuguese children, the cutoff was at least 48 [21,23]. There were additional non-scorable questions about the child’s wake time and bedtime on weekdays and on weekends, the total time of daily sleep, and whether the parents consider the child to have a sleep or falling asleep problem.

### 2.3. Statistical Analysis

The Shapiro–Wilk test was used to examine the normal distribution of the continuous variables. Levene’s test was used to evaluate homoscedasticity.

The sign test was used to compare the children’s wake-up hours and bedtimes on weekdays and at weekends. The Kruskal–Wallis test was used to compare wake time and bedtime and total hours of sleep by age group. The Mann–Whitney U and *t*-tests were used to compare the time the child wakes up, goes to bed, their total daily sleep time, and their results from the CSHQ-PT scales by sex, school area, electronic devices in the bedroom, single versus shared bedroom, existence of brothers/sisters in the family, dinner time, and PSA habits. The chi-square test of independence was used to analyze the association between the school area, the distance from home to school, and the time it takes the child to travel to school.

Logistic regression was used to examine the association of clinical factors with a deficit in the child’s total sleep time. The dependent variable was defined as y=1 if the parents reported that by day the total sleep time of the child was less than 10 h and y=0 otherwise. The explanatory variables considered were: the child’s characteristics (sex and age), the socioeconomic environment of the children (education of the person in charge of the education of the child, location area of the school, distance from the home to the school, single or shared bedroom, and electronic devices present in the bedroom), the daily habits of the child (schedules, PSA, and sleep habits) and sleep problems of the child (subscale scores of the CSHQ-PT). To fit the logistic regression model, we followed the approach suggested by Hosmer and Lemeshow [26]. The functional form of the continuous variables was evaluated by LOWESS and fractional polynomial methods. In the residual analysis, two observations were highlighted, as possible outliers. However, it was found that these observations had no significant impact on the adjusted model. The goodness of fit was tested using the Cessie–van Houwelingen and the Hosmer–Lemeshow tests. The final model presented a good fit, with an AUC value equal to 0.783.

R version 3.6.2 was used for statistical analyses. The level of significance was 5% (α=0.05).

## 3. Results

A little over half the children (51%) were male (Table 1). The age of the children varied between 5 and 11 years, with a mean age of 7.6 years (SD = 1.2 years) (Table 2).

Most (85.3%) of the children attended schools located in the urban area (with low population density); in total, 34.4% lived at least 3 km away from the school and 33.1% between 1 and 3 km. The distance between the child’s residence and the school was related to the school’s location. (p<0.001). There was a higher proportion of children living less than 1 km away from school among those attending a school in a rural area than in an urban area (45.1% vs. 30.3%). The opposite occurred when the child lived between 1 and 3 km away from school (21.1% vs. 35.2%). Students from urban schools arrived at school earlier, i.e., there were more students arriving at school before 8:30 a.m. (44.2% vs. 16.7%, p<0.001).

Most of the children lived with both parents (81.2%) and ¾ also live with brothers and sisters. The principal caregiver was almost always the mother (84.3%) or the father (14.5%). The most common level of education of the parents/guardians of the children studying in rural areas was secondary education (44.3%), while in urban areas, it was higher education (51.9%). Most of the parents/guardians were employed (79.7%), and only 12.3% were self-employed.

Most children (62.7%) slept in a single room. Almost half (48.7%) had electronic devices in the bedroom, usually a television (84.5%) and less frequently a mobile phone (10.7%) or other type of device (console, computers, tablets, etc.) (14.6%).

More than nine out of 10 children (91.3%) accumulated at least 60 min of PSA per day. Nine out of 10 parents reported that PSA helps children to sleep better.

### 3.1. Sleep Duration and Wake-Up and Bedtime Hours

Parents reported that, on average, the children slept 9 h and 37 min (SD = 44 min) per night, with 49.5% of children sleeping less than 10 h. The children woke up significantly earlier on weekdays than at the weekend (Me = 7:30 a.m. vs. 9:00 a.m., p<0.001), and also went to bed earlier on weekdays (Me = 9:30 p.m. vs. 10:30 p.m., p<0.001). There was a tendency for later bedtimes with increasing age both during weekdays (p<0.001 and weekends (p<0.001), as well as a reduction in sleep duration (p<0.001). At the weekend, female children woke up on average 17 min later than male children (Mean = 9:08 a.m. vs. 8:51 a.m., p<0.001) (Table 3).

The sleep duration for children attending schools in urban areas was significantly lower than for children attending schools in rural areas (Me = 9 h 38 min vs. 10 h, p=0.002). On weekdays, children attending schools in urban areas woke up earlier (Me = 7:30 a.m. vs. 7:55 a.m., p<0.001). No significant differences were found at bedtime on weekdays or weekends (both p>0.05).

Children who had a television in their bedroom slept less than those who did not (Me = 9 h 30 min vs. 10 h, p=0.045). The same was true for children who had other electronic devices in their bedroom (Me = 9 h 30 min vs. 10 h, p=0.008). Children who had electronic devices in their bedroom went to bed later than those who did not and, at the weekend, woke up later (all p<0.01). No significant difference was found in sleep duration between children sleeping in a single room and those sleeping in a shared room (p=0.564) or between children with or without brothers/sisters (p=0.892).

Children who had dinner before 8 p.m. slept longer than those who had dinner later (Me = 10 h vs. 9 h 30 min, p<0.001). Usually, they woke up and went to bed earlier than those who had dinner after 8 p.m. (all p<0.001).

Children who completed at least 60 min of PSA per day slept more hours per day than those who did not (Me = 10 h vs. 9 h 30 min, p<0.001) and went to bed significantly earlier (all p<0.05).

### 3.2. Cshq-Pt Sub-Scales and Global Scale

The prevalence rate of sleep disorders was 25.8%, although only 5.6% of parents considered their child to have sleeping or falling asleep problems.

A higher SDI was statistically associated with female children (Me = 44 vs. 43, p=0.006). Female children had higher scores in the subscales of sleep anxiety (p=0.012) and daytime sleepiness (p<0.001) (Table 3).

No significant difference was found in children’s SDI by the area where they attended school (p=0.767); however, children who attended a school in the urban area had a higher score on the daytime sleepiness subscale (Me = 13 vs. 12, p=0.025).

The SDI score was significantly higher in children who ate after 8 p.m. (Me = 44 vs. 43, p=0.002), as well as the score on the subscale of bedtime resistance (p=0.012), sleep duration (p<0.001), and daytime sleepiness (p<0.001).

The SDI score was significantly higher in children who had electronic devices in the bedroom (Me = 44 vs. 43, p=0.007), especially if the device was a mobile phone (Me = 46 vs. 43, p<0.001). These children tended to have higher rates in the subscales of sleep duration, parasomnias, and sleep-disordered breathing (all p<0.05, Table 3). The daytime sleepiness subscale score was statistically higher in children who had a cell phone (p=0.002) or other devices in the bedroom (p=0.011), and the score on the subscale sleep anxiety was higher for children with a television in the bedroom (p=0.007).

No significant differences were found in the SDI score for a cumulative time of at least 60 min of PSA per day (p=0.235), although the score for the parasomnia subscale was higher for children who accumulated at least 60 min of PSA per day (p=0.001). The SDI score was higher for children who belonged to a sports association than for the others (Me = 44 vs. 43, p=0.014), but the subscale of the bedtime resistance score was lower (p=0.008).

### 3.3. Factors Associated with Sleep Problems

According to the final logistic regression model, presented in Table 4, the factors that were revealed to be associated with a deficit in children’s sleep time (i.e., a sleep time of less than 10 h for children in primary school) were: being over 7 years old, living more than 3 km away from school, having electronic devices (other than television and mobile phone) in the bedroom, having a monthly expenditure under EUR 10 on PSA, going to bed after 9 p.m. on weekdays, arriving at school before 8:30 a.m., and having higher scores on the sleep duration and bedtime resistance subscales. Additionally, children who woke up before 7 a.m. on weekdays and ate dinner after 8 p.m. had: about 33 times more odds (OR CI95% = (7.45; 149.18)) than children who woke up after 7 a.m. and had dinner before 8 p.m.; almost 21 times more odds (OR CI95% = (4.62; 93.42)) than those who woke up after 7 a.m. and had dinner after 8 p.m.; and about nine times more odds (OR CI95% = (2.00; 44.24)) than those who woke up at 7 a.m. maximum and had dinner before 8 p.m.

## 4. Discussion

Socioeconomic level [8] and regular PSA [27] are associated with sleep habits. In addition, a bidirectional relationship between physical exercise and sleep has been assumed for some time, and both are associated with children’s health [28]. However, there are some contradictory or inconsistent findings in the literature. The main focus of interest of this study was the correlation between sleep habits, socioeconomic factors, and PSA in primary school children in a low-density population.

According to the parental reports, we observed that around half of the primary school children slept less than the recommended 10 h. On average, they had a deficit of 23 min of sleep time, which was more associated with older children. This value is similar to that obtained in another study (18 min) carried out in Portugal in 2014 [20]. In the Netherlands [29] and the United States of America [7], the mean sleep duration of children was higher than 10 h. In China, children slept an average of 35 min less than the recommended amount [7]. This difference in the number of hours of sleep remains in later ages, with adolescents in Asian countries sleeping 40 to 60 min less per night than those in the USA and 60 to 120 min less than Europeans [30]. The insufficient sleep time of children may be related to cultural and social habits [31].

The children of this study woke up and went to bed earlier on weekdays than on the weekend. Belmon et al. [14] found moderate evidence for the week’s schedule as a determinant of sleep timing on weekends. We found an age-effect for bedtime and sleep duration, with older children going to bed later, sleeping fewer hours, and with a higher risk of insufficient sleep time, which is in agreement with previous findings [23,32].

Some studies reported sex differences [7,33,34] and others no sex differences [35,36], with respect to sleep duration, sleep times, and sleep-related problems. We observed that female children woke up later on weekends and had higher sleep disorders, sleep anxiety, and daytime sleepiness. Similar results were observed in [25], where Dutch girls experienced more daytime sleepiness than boys [29]; however, this contradicts the higher daytime sleepiness observed in U.S. male children.

An earlier dinner time was associated with earlier wake-up and bedtimes, longer sleep duration (and less sleep deficit), and lower levels of sleep disorders, bedtime resistance, and daytime sleepiness. Sleep behaviour and mealtimes tend to coexist among young children [15,37]. Parents’ behaviours during bedtime and the night are the determinants of the sleep duration of young children [38]. Families should seek trained professionals to evaluate and intervene in sleep behaviours when there are persistent difficulties at this level [39].

As reported for Belgian children [33], children attending rural schools in Évora woke up later on weekdays. In addition, they presented a higher sleep duration, and daytime sleepiness was less common than children in urban schools. This result may be related to the children attending rural schools nearer the home than urban schools.

The presence of electronic devices was associated with adverse sleep outcomes for the children, such as later bedtimes, parasomnias, sleep-disordered breathing, daytime sleepiness, anxiety, and shortened sleep duration and insufficient sleep duration. This is consistent with previous studies on pediatric populations [22,40,41]. It is recommended to limit access to electronics during and after bedtime by removing electronics from children’s bedrooms [15]. The availability of electronic entertainment and communication devices allows children to use them after the time that their parents expect them to be asleep [42].

Children and adolescents should complete at least an average of 60 min per day of moderate to vigorous physical activity throughout the week [43]. Most of this physical activity should be aerobic. According to the World Health Organization guidelines, moderate to vigorous-intensity aerobic activities, as well as those that strengthen muscles and bones, should be incorporated at least 3 days a week.

In the surveyed schools, at least once a week there was physical and sports activity guided by teachers of curriculum enrichment activities. Students have a 30-min break for morning play and 30 min for afternoon play. In these 60 min, these children do free, spontaneous, unsupervised, non-oriented physical activity (moderate to vigorous). Parents’ responses were related to the knowledge of their children: they expected the child to be active during the breaks if their child was active.

In our study, children who accumulated at least 60 min of PSA per day went to bed earlier and slept more hours. Children who participated in or competed in PSA presented a lower level of sleep disorders but a higher bedtime resistance and a higher risk of sleep deficit. These findings could result from scheduled PSA. Competitive PSA requires a higher number of hours of training per day and, usually, training takes place after children leave school. Consequently, workouts can end close to bedtime, making it impossible for children to relax and establish a more restful state, contributing to bedtime resistance [15]. Furthermore, PSA improves sleep efficiency; therefore, a child engaged with more PSA may simply need fewer hours of sleep.

In this study, the prevalence of sleep disorders was 25.8% (i.e., SDI higher than cutoff), and only 5.6% recognized that there was a sleep problem. Parents’ underestimated perception has been identified previously in Portugal [23,36]. It can be explained by the social, cultural, and biological factors that contribute to how each parent perceives children’s sleep problems and what sleep practices are considered “normal” [19,44].

Compared to our study, in the 2013 Portuguese study, the prevalence rate was lower (10.4%), and no differences were found by population density areas [23]. However, higher scores were reported in the subscales of sleep duration and daytime sleepiness. Studies on the urban pediatric Portuguese population presented a higher incidence of sleep disorders (44% to 75%) [19,20], as well as higher subscales scores except sleep onset delay. This difference may be due to cultural and environmental differences between Portuguese regions. There are studies in other countries reporting differences in sleep problems between urban and rural school children [45,46,47].

This study had some limitations. Children’s sleep duration was obtained by the parent-reported questionnaire, which may differ from one obtained with objective measures, such as polysomnography or actigraphy. Sleep duration may differ between weekends and weekdays; however, this question was not asked separately for weekdays and weekends. The data were obtained in 2019, i.e., before the COVID-19 pandemic. Therefore, further research is needed to identify possible changes in children’s sleep habits due to the pandemic. This was a cross-sectional study. No causal relationship can be established between socioeconomic activity, physical activity, and sleep.

## 5. Conclusions

This research is important because it provides data and indications of the population of the municipality of Évora aged between 5 and 11 years old, regarding their sleep habits, their personal and socioeconomic characteristics, their daily PSA, and their daily routines.

It provides information about parents’ perception of children’s physical activity time and their financial investment in funding PSA, allowing for a reflection on local policies to support and encourage PSA.

The most important determinants for a perceived shorter children’s sleep time belonged to the socioeconomic determinants (i.e., being older and a longer distance between home and school), daytime and evening activities (i.e., the presence of electronic devices in the bedroom and engagement with competitive PSA), schedules (i.e., early arrival time at school, late bedtime, early wake-up, and late dinner time) and sleep disorders (sleep duration and bedtime resistance).

The study is also relevant because it allows not only for a reflection on local policies to support and encourage PSA but also for school health officials and pediatrics to carry out awareness campaigns for parents about sleep problems. On the other hand, the daily time given to the child to play is important because it relates to cognitive and motor development, the mental and physical health of the children, and is important for the monitoring of the public policies, impacts, and effects of decisions about children’s playtime.

Finally, to our knowledge, there are no studies that have analyzed these relationships in primary school children living in a low population density area of Portugal. There are some social, cultural, and biological differences between Portuguese regions. Therefore, it is important to investigate children’s sleep patterns among local communities for better health planning.

## Figures and Tables

**Table 1:** Sociodemographic characteristics of the children.

Characteristics	Categories	n	%
Sex	Male	722	51.0
	Female	695	49.0
School area	Urban	1227	85.3
	Rural	211	14.7
Distance from home to school	Up to 1 km	453	32.4
	1 to 3 km	463	33.1
	More than 3 km	481	34.4
Household	Both parents (with/without brothers)	1167	81.2
	One of the parents (with/without brothers/stepfather/stepmother)	242	16.8
	Without father and mother	29	2.0
Relationship of the caregiver	Father/Mother	1348	98.8
	Other	17	1.2

**Table 2:** Age and sleep habits of the children and scores of the CSHQ-PT (Min. = minimum, Q1 = first quartile, Q3 = third quartile, Max. = maximum, SD = standard deviation, n = number of valid observations). For each subscale of the CHSQ, the numbers in parenthesis are the possible minimum and maximum scores.

Characteristics	Min.	Q1	Median	Mean	Q3	Max.	SD	n
Age (years)	5.0	7.0	7.0	7.6	9.0	11.0	1.2	1436
Weekday wake-up time	6:00	7:20	7:30	7:35	8:00	10:00	00:25	1424
Weekend wake-up time	6:00	8:30	9:00	9:00	9:30	12:30	00:53	1406
Weekday bedtime	19:00	21:30	21:30	21:36	22:00	23:30	00:28	1387
Weekend bedtime	20:00	22:00	22:30	22:27	23:00	01:00	00:38	1375
Total sleep time per day (h)	6.5	9.0	10.0	9.6	10.0	12.0	0.74	1379
Subscale/Scale								
Bedtime resistance (6–18)	6.0	6.0	7.0	7.8	9.0	17.0	2.3	1381
Sleep onset delay (1–3)	1.0	1.0	2.0	2.0	3.0	3.0	0.9	1428
Sleep duration (3–9)	3.0	3.0	3.0	3.6	4.0	9.0	1.0	1384
Sleep anxiety (4–12)	4.0	4.0	5.0	5.4	6.0	12.0	1.7	1392
Night wakings (3–9)	3.0	3.0	3.0	3.6	4.0	9.0	1.0	1392
Parasomnias (7–21)	7.0	7.0	8.0	8.6	9.0	19.0	1.6	1393
Sleep-disordered breathing blue (3–9)	3.0	3.0	3.0	3.4	3.0	9.0	0.9	1419
Daytime sleepiness (8–24)	8.0	11.0	13.0	12.9	15.0	23.0	2.8	1361
Sleep disturbance index	33.0	40.0	44.0	44.5	48.0	70.0	6.0	1182

**Table 3:** *p*-value of the Mann–Whitney U test (or *t*-test) for wake-up and bedtime on weekdays and on weekends, total sleep time, and the scores of the CSHQ-PT by sex, area where the school is located (school area), electronic devices present in the bedroom, dinner time before or after 8 p.m. (dinner time), and PSA practice (≥60 min. = the child accumulates at least 60 min of PSA per day; Fed./Comp. = the child belongs to a sports association).

Variables	Sex	School Area	Electronic Devices in the Bedroom				Dinner Time	PSA	
			Yes	TV	Mobile Phone	Others		≥60 min.	Fed./ Comp.
Weekday wake-up time	0.469	<0.001	0.423	0.215	0.454	0.026	<0.001	0.457	0.491
Weekend wake-up time	<0.001 ^a^	0.288 ^a^	<0.001 ^a^	<0.001 ^a^	0.029 ^a^	0.915 ^a^	<0.001 ^a^	0.892 ^a^	0.357 ^a^
Weekday bedtime	0.685	0.465	0.006	0.015	0.299	0.742	<0.001	0.018	0.766
Weekend bedtime	0.801	0.995	<0.001	<0.001	0.564	0.017	<0.001	0.041	0.305
Total hours of sleep per day	0.113	0.002	0.006	0.045	0.225	0.008	<0.001	<0.001	0.880
Subscale/Scale									
Bedtime resistance	0.063	0.097	0.430	0.352	0.495	0.276	0.012	0.355	0.008
Sleep onset delay	0.677	0.256	0.275	0.504	0.888	0.561	0.982	0.170	0.992
Sleep duration	0.629	0.451	0.003	0.002	0.032	0.557	<0.001	0.155	0.121
Sleep anxiety	0.012	0.950	0.005	0.007	0.131	0.384	0.328	0.514	0.164
Night wakings	0.741	0.767	0.763	0.482	0.920	0.372	0.940	0.904	0.278
Parasomnias	0.310	0.119	0.001	<0.001	0.022	0.794	0.816	0.001	0.423
Sleep-disordered breathing	0.651	0.448	<0.001	0.002	0.039	0.053	0.816	0.157	0.116
Daytime sleepiness	<0.001	0.025	0.196	0.889	0.002	0.011	<0.001	0.926	0.857
Sleep disturbance index	0.006	0.876	0.007	0.051	<0.001	0.849	0.002	0.235	0.014

^a^*t*-test

**Table 4:** Adjusted logistic regression model for the insufficient sleep time of the children. In this table, we present the coefficients of the model, the standard deviations, the *p*-values, the odds ratio (OR), and the 95% confidence interval obtained from the Wald statistic.

Variables	Coefficient	Std. Error	*p*-Value	OR	OR CI95%
Constant	−3.137	0.592	<0.001		
Sex (ref.: Female)					
Male	0.273	0.155	0.077	1.31	(0.97; 1.78)
Age (ref.: <=7 years old)					
>7 years old	−0.406	0.153	0.008	0.67	(0.49; 0.90)
Distance from home to school (ref.: >3 km)					
Up to 1 km	−0.772	0.191	<0.001	0.46	(0.32; 0.67)
1 to 3 km	−0.347	0.183	0.058	0.71	(0.49; 1.01)
Electronic devices in the bedroom (ref.: No)					
Yes	0.670	0.283	0.018	1.95	(1.13; 3.43)
Belong to a sports association (ref.: No)					
Yes	0.297	0.171	0.083	1.35	(0.96; 1.88)
Monthly expenditure on PSA (ref.: <EUR 10)					
>=EUR 10	−0.374	0.185	0.043	0.69	(0.48; 0.99)
Weekday wake-up time (ref.: After 7 a.m.)					
Before 7 a.m.	1.265	0.277	<0.001		
Weekday bedtime (ref.: Before 9 p.m.)					
After 9 p.m.	1.557	0.256	<0.001	4.74	(2.91; 7.97)
Dinner time (ref.: After 8 p.m.)					
Before 8 p.m.	0.472	0.164	0.004		
Arrival time at school (ref.: Before 8:30 a.m.)	−0.837	0.162	<0.001	0.43	(0.32; 0.59)
After 8:30 a.m.					
Bedtime resistance subscale	0.126	0.034	<0.001	1.13	(1.06; 1.21)
Sleep duration subscale	0.466	0.096	<0.001	1.59	(1.33; 1.93)
Daytime sleepiness subscale	0.050	0.029	0.087	1.05	(0.99; 1.11)
Weekday wake-up time × Dinner time					
Before 7 a.m. × Before 8 p.m.	1.770	0.805	0.028		

Cessie–van Houwelingen test: *p* = 0.814; Hosmer–Lemeshow test: *p* = 0.5; sensibility = 74%; specificity = 69.2%; cutoff point = 0.485: AUC = 0.783.

## Data Availability

Not applicable.

## Abbreviations

The following abbreviations are used in this manuscript:

AASM	American Academy of Sleep Medicine
CSHQ-PT	Portuguese version of the Children’s Sleep Habits Questionnaire
PSA	Physical and sports activity
SDI	Sleep disturbance index
